# Voluntary Food Reformulation Initiatives Failed to Reduce the Salt Content of Artisanal Breads in Greece

**DOI:** 10.3390/nu17213374

**Published:** 2025-10-27

**Authors:** Georgios Marakis, Sotiria Kotopoulou, Stavroula Skoulika, Georgios Petropoulos, Zoe Mousia, Emmanuella Magriplis, Antonis Zampelas

**Affiliations:** 1 Hellenic Food Authority, 11526 Athens, Greece; gmarakis@efet.gr (G.M.); 2 Department of Food Science and Human Nutrition, Agricultural University of Athens, 11855 Athens, Greece; 3 Department of Life Sciences, School of Life and Health Sciences, University of Nicosia, CY 2417 Nicosia, Cyprus

**Keywords:** salt, artisanal bread, MoU, WHO sodium benchmarks, modeling scenarios

## Abstract

Background: Reducing salt in bread is considered a straightforward, cost-effective public health intervention and is implemented in several countries, either voluntarily or through legislation. A Memorandum of Understanding (MoU) was signed in Greece in 2016, setting a voluntary maximum salt content of 1.2% in artisanal bread. This study aimed to evaluate the effectiveness of the MoU and assessed the potential impact of reducing salt in bread on overall salt intake, using the MoU target and the relevant WHO global sodium benchmark. Methods: Artisanal bread samples (*n* = 253) randomly collected from different parts of Greece in 2024 were analyzed for salt content and compared with samples collected in 2012 (*n* = 220). Salt intake from bread was estimated using data from the Hellenic National Nutrition and Health Survey (HNNHS), and modeling scenarios were conducted. Results: The MoU and related voluntary awareness activities were ineffective as a strategy to reduce salt in bread. The mean salt content in bread in 2024 was 1.41 (0.30)%, representing a 6.8% increase compared to 1.32 (0.31)% in 2012. Only 19.4% of samples in 2024 contained ≤1.2% salt, compared to 31.8% in 2012. Full MoU compliance would enable an additional 3.1% of Greek bread consumers, currently exceeding 5 g in their daily salt intake from foods alone, to reduce their intake to below 5 g. This would rise to 6.2% if the WHO sodium benchmark was implemented. Conclusions: A mandatory salt limit, aligned with the WHO global benchmark, is urgently needed to support national reformulation strategies. This work can contribute to European and international discussions on food reformulation.

## 1. Introduction

Excessive salt consumption is recognized by the World Health Organization (WHO) as one of the most important dietary risk factors of non-communicable diseases worldwide, as it increases the risk of health conditions, including hypertension [1,2]. Evidence indicates that reducing salt intake significantly lowers both systolic and diastolic blood pressure without adverse effects [3] and contributes to the prevention of major cardiovascular events, particularly in low-cardiovascular-risk populations [4]. Despite these serious health concerns, salt, chemically defined as sodium chloride, plays an important role in food manufacturing and preservation. Nevertheless, salt intake reduction is regarded as one of the most cost-effective preventive public health interventions to address the growing burden of non-communicable diseases [5,6]. WHO Member States have committed to reducing the average population’s sodium intake by 30% by the year 2030 [7]. However, no country was on track to meet this target as of 2023 [7]. A key pillar for reducing population-level salt intake is food reformulation [8], particularly of foods that contribute the most to salt intake. To support national policy actions, WHO has developed and recently updated global sodium benchmarks across various food categories [2]. Modeling studies have demonstrated that implementing these benchmarks could yield substantial health and economic benefits in both high-income [9] and lower-middle-income countries [10].

Currently, salt intake in Greece [11,12] is almost double the target set by the WHO in its 2012 guidelines [13], and is comparable to the average salt intake globally [7]. Salt intake in Greece does not appear to be related to adherence to the Mediterranean dietary model [11] or to the population’s salt-related knowledge [12], suggesting that most salt consumption comes from processed foods. According to the latest Hellenic National Nutrition and Health Survey (HNNHS) (2013–2015), processed cereal products, including bread, are the leading contributor to salt intake among adults in Greece [14], without taking into account salt added during cooking or at the table; this is a common finding in many developed and developing countries [15,16,17,18,19], especially where bread is consumed daily [20]. Reducing salt content in cereal products, particularly bread, can lower daily sodium intake, potentially decreasing cardiovascular events and, mainly, stroke. Results from an Irish intervention trial showed that a pragmatic reduced-salt diet incorporating low-salt bread (0.3% salt) reduced salt intake by 1.7 g/day, with corresponding significant and clinically meaningful decreases in systolic blood pressure [21]. Additionally, Ferrante et al. (2011) [22] reported that substituting bread containing 2% salt with lower-salt bread (1.4% salt) significantly reduced systolic blood pressure by 1.66 mmHg and diastolic blood pressure by 0.76 mmHg.

Following the voluntary EU Framework for National Salt Initiatives and the adoption of the European Council conclusions on 8 June 2010 of ‘*Action to reduce population salt intake for better health*’ [23], the Hellenic Food Authority developed the Salt Reduction Strategy 2016–2020, which was then included in Greece’s National Action Plan for Food Reformulation [24]. In 2016, the Hellenic Food Authority (EFET) signed a Memorandum of Understanding (MoU) with the Hellenic Federation of Bakers to reduce salt content in all bread (excluding specialty breads containing cheese, olives and/or sundried tomatoes). This MoU set an upper 1.2% salt limit target for bread (as consumed) on a voluntary basis. This target was proposed by the Federation, based on bread composition data from the official control program of the Hellenic Food Authority, conducted in 2012, and on practical feasibility, and was applied specifically to non-prepackaged artisanal bread, which is the most commonly consumed bread type in Greece [25]. Additionally, this initiative was supported by targeted communication efforts to bakers, including an article in a bakers’ magazine, events dedicated to raising awareness about the MoU and personal letters to the President of Bakers in each regional unit, emphasizing the importance of gradual salt reduction in bread. In addition, communication efforts by the Hellenic Food Authority, the Consumers Association “EKPOIZO” and the Hellenic Association of Dieticians and Nutritionists raised public awareness and encouraged consumers to seek bread with reduced salt content. To date, no monitoring or evaluation of this voluntary initiative has been undertaken.

This study aimed to (a) assess whether the voluntary salt reduction initiative has met its primary goal, (b) assess the impact of salt reduction in bread on salt intake (modeling scenarios) using consumption data from the HNNHS, the MoU target and the WHO global sodium benchmark for leavened bread and (c) discuss challenges and suggest future actions. This work aims to provide useful insights into the effectiveness of voluntary reformulation strategies and inform the debate on whether mandatory sodium limits may be more appropriate.

## 2. Methodology

### 2.1. Bread Sampling

Data of the salt content in bread from two sampling periods (2012 and 2024) were utilized in this work. The sampling procedure in 2012 has previously been described in a technical report by the Hellenic Food Authority [26]. A total of 220 bread samples were collected from two regions in Greece: Attica (*n* = 111) and Epirus (*n* = 109), from bakeries located in both urban and rural areas. According to the 2011 Census, these regions covered 38.5% of Greece’s population. Sampling ran from May to November 2012 and, in the absence of national dietary survey data, data on bread consumption patterns by the Hellenic Statistical Authority (EL.STAT.) guided sampling. Two-thirds of the samples included bread made with refined wheat flour and refined durum wheat flour. The remaining samples were made with whole-wheat flour, mixtures of whole-wheat and refined wheat, or breads made with rye flour. All bread samples were non-prepackaged and collected randomly from different bakeries by Hellenic Food Authority inspectors.

In 2024, 253 bread samples were collected by Hellenic Food Authority inspectors from bakeries in 9 regions [Attica (*n* = 146), Central Macedonia (*n* = 40), North Aegean (*n* = 5), eastern Macedonia and Thrace (*n* = 25), Thessaly (*n* = 5), Peloponnese (*n* = 6), West Greece (*n* = 6), Epirus (*n* = 10) and Crete (*n* = 10)]. These regions covered approximately 87.5% of Greece’s population, according to the 2021 census. Sampling ran from March to May 2024. Samples were also collected randomly from bakeries in both urban and rural areas. Bread types included the following: white bread made with refined wheat flour, bread made with refined durum wheat flour, wholemeal bread, wholemeal bread with seeds and rye bread, as well as specialty breads, such as bread made with corn and wheat flours, bread made with Dinkel flour, bread with walnuts, low glycemic index bread and bread with no added salt) (see Supplementary Table S1). In this sampling plan, approximately half of the samples were bread made with refined wheat flour.

### 2.2. Determination of Sodium Content in Bread

Sodium content in bread collected in 2012 was determined by the General Chemical State Laboratory (GCSL) of Greece. A concise report of the results is available on the Hellenic Food Authority website [26]. Preliminary analyses indicated artisanal bread loaves were homogenous in terms of salt distribution. Hence, two thin slices (2–3 mm thick) were cut from different sections of each loaf and used for sodium quantification. The slices were immediately weighed by using an analytical balance with a precision of 0.1 mg (Precisa 205A, Dietikon, Switzerland), then dried at 60 °C for 8 h in an oven (Memmert UNE400, Schwabach, Germany) until a constant weight was achieved. After cooling to room temperature using a desiccator, the slices were weighed again to calculate moisture content and convert the sodium concentration measured in dried samples to the concentration in the bread as sold. Dried slices were ground and homogenized using a household grinder. A quantity of 0.15–0.20 g from the homogenized samples was weighed in 20 mL glass vials with 0.1 mg accuracy, and 3 mL of ultra clean nitric acid (Fisher Chemicals TraceMetal Grade, Pittsburgh, PA, USA, 67–69% HNO_3_) was added. Each sample was processed in duplicate. Vials were kept at 40–50 °C overnight, until full digestion of the bread matrix was achieved. The digested solution was transferred to 100 mL volumetric flasks with water. Sodium concentration in the solution was determined by flame photometry (Flame photometer Jenway PFP7, Essex, UK) and converted to sodium concentration. The final sodium content of the bread samples as sold was calculated as the average value of the duplicate analyses. FAPAS test material 1864 was included in each analytical batch for quality control.

The samples collected in 2024 were analyzed at the premises of the Hellenic Food Authority labs in Athens. Sodium content was determined using an in-house method, based on EN 15505:2008 [27]. Briefly, 0.45–0.55 g of each homogenized sample was digested with 8 mL HNO_3_ (>68%, Nitric acid S.G. 1.42, Fisher Chemicals, Loughborough, UK) in an Anton Paar Multiwave 3000 (Graz, Austria) microwave oven. The digested samples were transferred to 50 mL volumetric flasks, and after the addition of 0.5 mL of Lanthanum standard solution (1000 μg/mL in 5% HNO_3_, VHG, Manchester, NH, USA), were filled up to 50 mL with de-ionized water. Each digestion batch included blank reagent (HNO_3_) and certified reference material (TYG077RM, Fapas, York, UK) to ensure reliable measurements. Sodium standard solution (1.000 g/L, PanReac AppliChem, Darmstad, Germany) was used to prepare working standard solutions of 0.20, 0.40, 0.60, 0.80, 1.00 and 1.50 mg/L. Lanthanum was added to a concentration of 1%, to eliminate the ionization interferences. The sodium content was determined by Flame Absorption Spectrometry (Na: 589 nm, slit: 0.2 nm, gas: air–acetylene) on a Shimadzu AA-6300 Atomic Absorption Spectrophotometer (Kyoto, Japan).

Results in salt content values (g/100 g) were classified into predefined ranges, <0.5, 0.5–1, 1.01–1.2, 1.21–1.5, 1.51–2 and >2.01, retained from the previous study [26] to allow comparisons between 2012 and 2024 and to reflect public health benchmarks and regulatory targets. Finer categorization within the 1.01–2 g/100 g range enabled the detection of shifts in the most frequently observed salt content intervals.

### 2.3. Hellenic National Nutrition and Health Survey Design and Study Population

This study included participants from the Hellenic National Nutrition and Health Survey (HNNHS), which surveyed a nationally representative sample of non-institutionalized, non-pregnant, and non-breastfeeding Greek adults. HNNHS was carried out from 1 September 2013 to 31 May 2015. Stratification was performed according to: (a) geographical density criteria by Greek region (7 regions), as provided by the Hellenic Statistical Authority; (b) age group; and (c) gender distribution. The study was conducted in accordance with the Declaration of Helsinki, and was approved by the Agricultural University of Athens Ethics Committee (No: 743/31-05-2013), with all participants providing written informed consent. The detailed methodology and questionnaires from the HNNHS are available in previous publications [28]. To date, no further national dietary surveys have been conducted.

**Figure 1:**
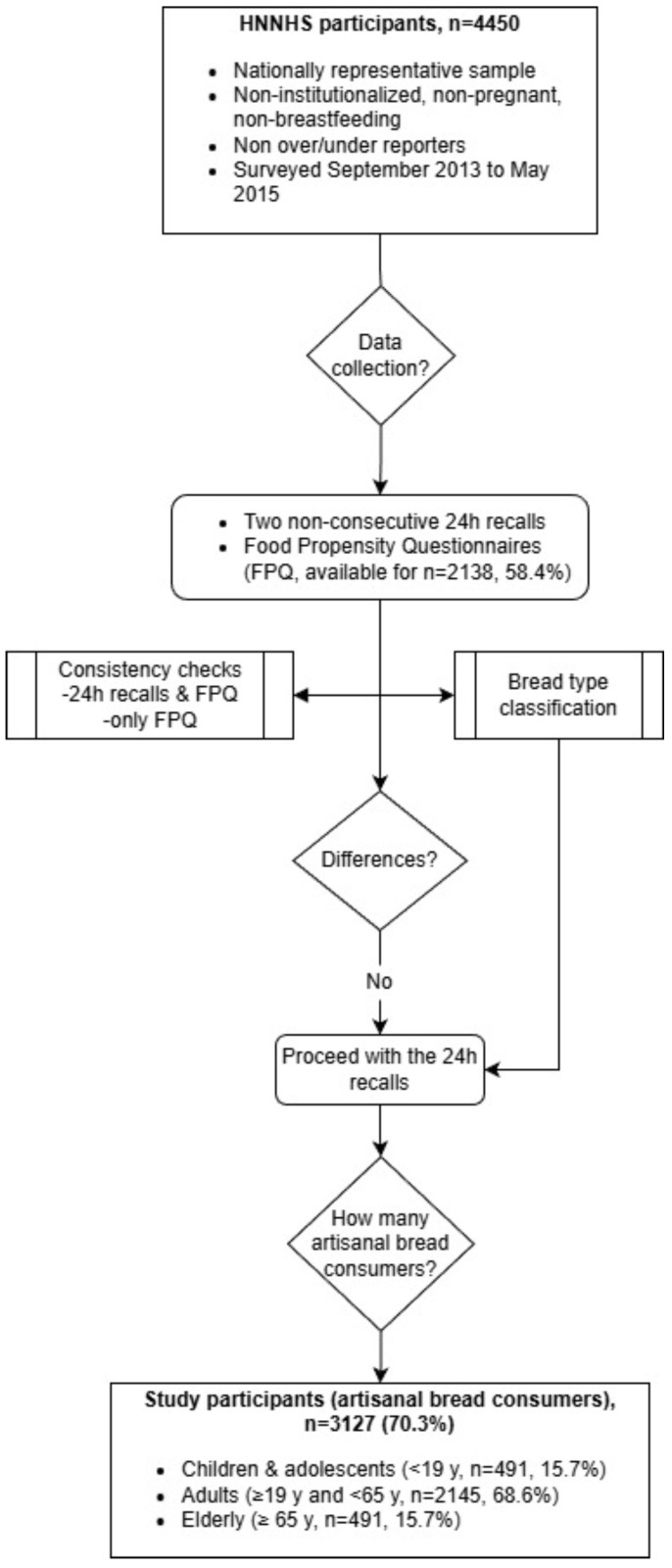
Study participant flowchart.

Extreme over- and under-reporters (defined as individuals reporting >6000 kcal/day and <500 kcal/day, respectively) were excluded from the analysis. A total of 4450 individuals across all age groups were enrolled (57.5% females) (henceforth: general population). Data of 3127 individuals (70.3% of population, 55.3% females) consuming artisanal bread (henceforth: consumers) were retrieved and classified in the age groups: children and adolescents (<19 years), adults (≥19 years and <65 years) and elderly (≥65 years), as depicted in Figure 1—Participant Flowchart.

### 2.4. Artisanal Bread Consumption Quantification

Food consumption data were recorded through two non-consecutive 24 h dietary recalls, 8–20 days apart, by trained professionals, using the Automated Multiple Pass Method. Details have been published previously [29]. Artisanal bread intake (whole, partial or in recipes) was calculated in grams per individual and averaged over the two 24 h dietary recalls. The HNNHS database was also updated to classify bread types into two broad categories, white and brown, based on flour type, and further for the categories classified used in 2012 presentation of results [26], as shown in Supplementary Table S1.

To evaluate the consistency of bread intake quantification before proceeding with salt intake estimation and modeling, a comparative analysis was conducted using the bread consumption frequencies reported in the frequency propensity questionnaire (FPQ) administered in the HNNHS. Details on the FPQ are provided elsewhere [30].

FPQ data were available for 2138 out of 3127 bread consumers (68.4%) enrolled in our study. For the remaining 989 consumers (31.6%), only the quantified artisanal bread consumption from the 24 h recalls was included in the analysis.

Daily bread intake was also calculated by multiplying consumption frequency with reported amounts. For individuals reporting both refined white and brown bread consumption (*n* = 1975), the average frequency was used with the total bread amount (consumption quantification scenario 1.1). When only one bread type was reported, its specific frequency was applied (consumption quantification scenario 1.2). When no frequency was reported, daily amounts were retained as is. Additionally, individual daily bread intake was determined by summing the frequencies of refined white and brown bread consumption and applying the same assumptions for single-type reports or missing frequency data (consumption quantification scenario 1.3).

Furthermore, estimated grams of refined and whole wheat bread consumption were calculated based solely on responses to FPQ (consumption quantification scenario 2). FPQ data on refined and/or whole wheat bread consumption were available for 3069 respondents (89.7% of all HNNHS participants). The methodology applied to estimate quantities of bread consumed was consistent with that described in the previous paragraph.

Consumption quantification scenarios are shown in Supplementary Table S2 and depicted in Figure 1—Participant Flowchart. Given that no considerable differences were observed in the cross-check comparative analyses regarding the median quantified grams of bread consumed, and that the use of FPQ data involved additional assumptions, we proceeded with the analyses to estimate salt intake and modeling scenarios using artisanal bread intake data from the two 24 h dietary recalls, without using FPQ data (consumption quantification scenario 1).

### 2.5. Salt Intake Quantification

The percentages of mean salt contents from 2012 and 2024, as shown in Supplementary Table S1, were used to update the HNNHS database to estimate salt intakes, sampled for the substitution models. For 2012, already-calculated mean salt contents per bread type were used [26]. For 2024, the mean sodium content was converted to the mean salt content using a conversion factor of 2.5. Two models were generated using data obtained at different times. Keeping artisanal bread consumption constant (the assumption of no changes in intake was made for substitution reasons), but modifying salt content as measured, differences were calculated by comparing 2024 to 2012 assessments.

To calculate the salt intake per artisanal bread-eating event, the following formula was applied:

$$salt\;intake\left(g\right)=\frac{salt\;content\;per\;100\;g\;of\;product\;x\;amount\;of\;bread\;consumed\;(g)}{100\;g\;of\;bread}$$

For ciabatta bread, which was not sampled in 2024 but was consumed in the HNNHS (*n* = 7), the analytical sodium content value from the 2012 sampling was used (1.68 g salt/100 g). For each individual, the salt intake from all artisanal bread consumption events was summed and averaged across the two 24 h dietary recall periods. Other dietary composition and intakes were also expected to remain unchanged through this procedure.

### 2.6. Modeling Scenarios About Salt Reduction

We estimated the total reduction in salt intake from bread and from all foods if all artisanal bread consumed complied with various maximum salt limits. Two counterfactual salt reduction scenarios were modeled:

Counterfactual Scenario 1

Total reduction in salt consumption was estimated, assuming that all artisanal bread would comply with the MoU target of 1.2 g of salt per 100 g of final product. If the mean salt content was ≤1.2 g/100 g, the existing value was retained; if exceeded, it was replaced with 1.2 g/100 g.

Counterfactual Scenario 2

The total reduction in salt consumption was estimated, assuming that all artisanal bread would comply with the WHO global sodium benchmark for leavened bread, set at 370 mg of sodium per 100 g [2]. This benchmark was converted to salt by multiplying by 2.5 and dividing by 1000, resulting in a target of 0.925 g of salt per 100 g of final product. If the mean salt content was ≤0.925 g/100 g, the existing value was retained; if exceeded, it was replaced with 0.925 g/100 g. The same benchmark was chosen for Lagana, as it is classified as a leavened bread due to the use of yeast or sourdough, although it may be considered a flatbread.

Finally, since total daily salt intake from all foods was recorded in the HNNHS database, these values were updated by replacing previous estimates of bread-derived salt intake with the newly calculated values. Subsequently, the percentage of individuals exceeding or remaining below the WHO recommended threshold of 5 g/day of salt intake from foods only (excluding discretionary salt use during cooking or on the plate) was estimated across all scenarios.

### 2.7. Sensitivity Analysis

To account for potential regional differences between the sampling years, a sensitivity analysis using data from the two prefectures that were sampled in both 2012 and 2024 was conducted.

### 2.8. Statistical Analysis

Baseline variables were stratified by age group (children and adolescents, 0–18 years | adults, 19–64.99 years | elderly, ≥65 years), to identify statistically significant differences between intakes (*p*-value, *p*-trend value). The selection of age groups was made to align with the classification used in the National Dietary Guidelines [31], while also ensuring sufficient analytical power.

Percentage changes in the mean salt contents between 2012 and 2024 were calculated using the following formula:

$$\%change=\frac{Mean2024-Mean2012}{Mean2012}\times 100$$

The distribution of median daily bread intake across the 9 Greek prefectures was calculated by dividing the median daily bread intake of each prefecture by the sum of the median daily intakes of all prefectures, and then multiplying by 100 to express the result as a percentage contribution.

The standard deviation (SD) was also calculated from the standard error of the mean (SEM), following the approach presented in previous research [1] to enable a comparison of results, using the formula:

SD=SEM√n

Means (standard deviations, sd) were used to describe normally distributed continuous variables and medians (25th and 75th percentiles) for skewed distributions. Categorical variables were expressed as frequencies, and between-group distribution differences were examined using chi square for proportions. ANOVA or the Kruskal–Wallis rank sum test were used for continuous data, depending on distribution. The Wilcoxon signed-rank test checked statistically significant differences in salt intake between 2012 and 2024. Statistical analysis was performed using STATA 13.0 (Texas Ltd., College Station, TX, USA).

## 3. Results

### 3.1. Salt Content Levels in Bread in 2024 to Those in 2012 (Prior to the MoU)

The mean (sd) salt content in artisanal bread samples in 2024 was 1.41 (0.30) g per 100 g, representing a 6.8% increase compared to 1.32 (0.31) g per 100 g in 2012. Table 1 presents the mean salt content of white and brown bread for both sampling years, and the percentage change. Salt content increased in all bread types, ranging from 5.8% in white bread to 8.1% in brown bread.

**Figure 2:**
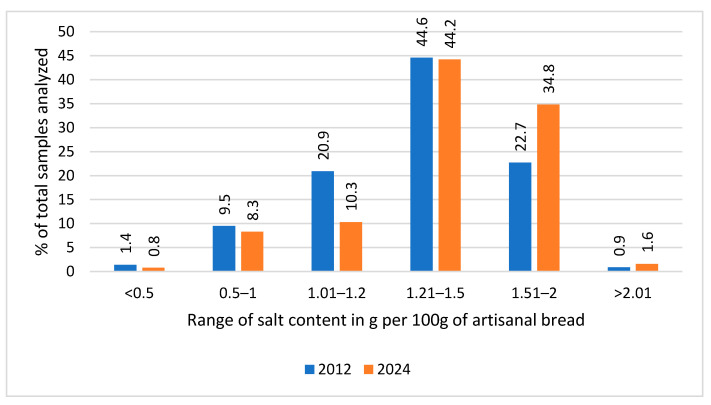
Percentage distribution of bread product samples across predefined salt content ranges (g/100 g) in 2012 and 2024.

Sample distribution across salt content ranges changed in 2024, compared to 2012. Distribution across ranges for both years appears in Figure 2, which highlights notable increases in the higher salt content ranges in 2024. Specifically, a 77.8% increase in samples exceeding 2.01 g/100 g of product and a 53.3% increase within 1.51–2 g/100 g were observed. Only 19.4% of samples in 2024 fell under 1.2 g/100 g of bread, compared with 31.8% in 2012 (39% reduced compliance). Supplementary Table S3 shows detailed percentage shifts.

### 3.2. Bread Intake Quantification Based on HNNHS Consumption Data

Baseline characteristics of the study participants are presented in Supplementary Table S4. Daily median artisanal bread consumption was 33 g (IQR: 19.8, 55), with high consumers (95th percentile—p95) reaching 118 g, and the overall range was 0.5–436 g. Supplementary Table S2 shows artisanal bread consumption estimates, as described in the Section 2. The average artisanal bread consumption, considering frequency, was 32.8 g, similar to the 33 g estimated using the 2 × 24 h recalls.

**Figure 3:**
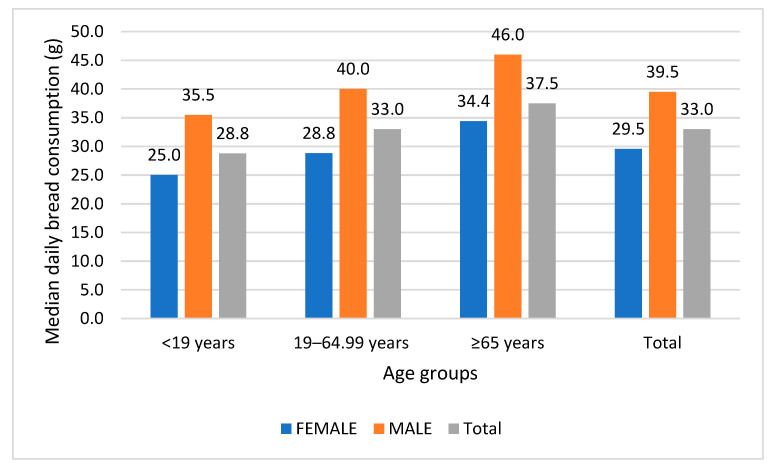
Median daily artisanal bread consumption by gender and age group (g).

Daily consumption was higher in males, with a median of 39.5 g (23.5, 66.0) vs. females with a median 29.5 g (16.5, 49.2). The median daily bread consumption was higher in the elderly (≥65 years) with 37.5 g (25, 66) per day (Figure 3).

### 3.3. Salt Intakes from Artisanal Bread Consumption in 2012 and 2024 Using Substitution Models

**Figure 4:**
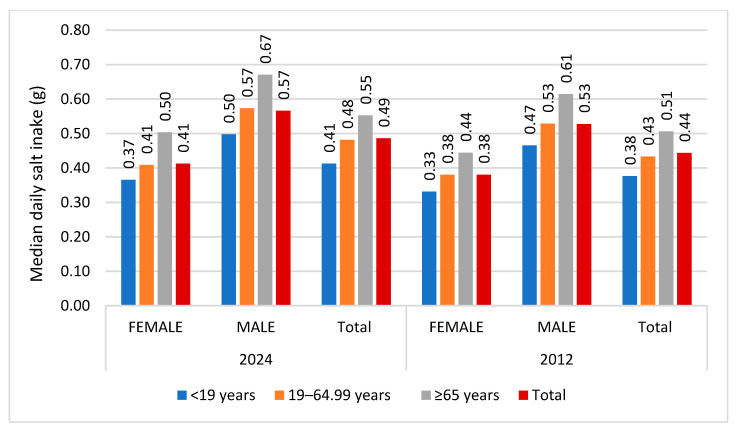
Median daily salt intakes (g) from artisanal breads in 2024 and 2012 by gender and age group.

The median daily salt intake from the artisanal bread was 0.49 g (IQR: 0.27, 0.80) in 2024 vs. 0.44 g (IQR: 0.25, 0.73) in 2012, representing a statistically significant increase of 11.4% (*p* < 0.05). Figure 4 presents the median daily salt intakes by gender and age group. Increases were observed in both females and males (7.9% and 7.5%, respectively), and in all age groups (<19 years, 19–64.99 years and ≥65 years by 7.9%, 11.6% and 7.8%, respectively).

The sensitivity analysis restricted to data from Attica and Epirus, the two prefectures where samples were collected in both 2012 and 2024, showed a median salt intake of 0.47 g (IQR: 0.24, 0.78) in 2024, which was also higher than the corresponding 2012 estimate (0.44 g; IQR: 0.25, 0.73). This indicates increased salt intake over time and confirms that the broader 2024 sampling did not affect the results (see Supplementary Table S5).

### 3.4. Modeling Salt Intake Reduction Scenarios

Utilizing the 2024 bread salt content data, the median daily salt intake from artisanal bread alone was estimated to be 0.49 g, accounting for 9.5% of the median total daily salt intake from all foods. Table 2 presents the impact of salt reduction counterfactual scenarios, as described in the Section 2, on the median daily salt intake from artisanal bread, total salt intake, and number and percentage of consumers exceeding the WHO limit of 5 g for daily salt intake. At baseline, 55.13% of consumers (1724 of 3127) exceeded the WHO salt intake limit from foods only (i.e., excluding discretionary salt use).

If all artisanal bread complied with the MoU maximum salt threshold of 1.2 g/100 g (counterfactual scenario 1), the estimated daily bread-related salt intake would decrease by 0.10 g to 0.39 g per consumer. This would reduce total daily median salt by 0.13 g to 5.18 g per consumer, reducing the exceeders of 5 g daily salt intake by 55, corresponding to a 1.7% reduction among bread consumers overall and a 3.2% reduction relative to baseline exceeders.

Similarly, if all artisanal bread products met the stricter WHO threshold of 0.925 g/100 g bread (counterfactual scenario 2), the median daily bread-related salt intake would drop by 0.18 g to 0.31 g per consumer. This would reduce the total daily median salt intake to 5.09 g, with 108 fewer consumers exceeding the WHO threshold, representing a 3.4% reduction among bread consumers overall and a 6.3% reduction relative to baseline exceeders.

## 4. Discussion

This study documents that the voluntary salt reduction initiative, launched through MoU between the Hellenic Food Authority and the Hellenic Bakers Federation in 2016, and related awareness activities were found to be ineffective in reducing salt content in artisanal bread. Contrary to the voluntary agreement and the communication measures undertaken to support it, a 6.8% salt increase was observed in 2024 compared to 2012.

Our findings diverge notably from those in other European countries, such as Slovenia, the United Kingdom, Ireland and the Netherlands, where similar voluntary initiatives achieved measurable decreases, albeit to differing extents. In Slovenia, where the bread sampling periods (2012 and 2022) were comparable to those in Greece (2012 and 2024), the average salt content declined from 1.35% to 1.26%, reflecting a 7% decrease [32]. In the Netherlands, the salt content in bread was reduced from 1.29% in 2011 to 1.04% in 2016 (9% decline) [33]. Similarly, in the UK, the average salt content in bread declined from 1.23% in 2001 to 0.98% in 2011 (18% decrease) [16], while in Ireland, the average salt content in white bread decreased from 1.4% in 2003 to 1.1% in 2011, representing a 21% reduction [21].

This study focused on assessing the effectiveness of the MoU, without analyzing the factors that contributed to its failure to produce impact. Among the factors that might have contributed to this disappointing outcome is the decline in bread purchases in Greece, as documented by the Hellenic Statistical Authority [34,35], which might have influenced producers’ willingness to modify recipes. Specifically, bakers might have been reluctant to reduce salt due to concerns about potential adverse effects on consumer acceptability, which could cause a further loss of customers and diminished profits. This apprehension aligns with findings from Kuhar et al. (2020) [36], who reported that in highly competitive markets, food producers often perceive salt reduction as risky, fearing that reformulated products may fail to meet consumers’ sensory expectations, ultimately leading to decreased sales, despite evidence indicating otherwise [37]. However, Lobo and Ferreira (2021) [38] identified 0.98% as the optimal salt content in bread from a hedonic (consumer preference) perspective, which was substantially lower than the levels found in the majority of bread sold in Greece. Furthermore, consumer attitudes might have also played a role. In a survey conducted in 2011, a significant proportion of the Greek population did not perceive their salt intake as excessive, and only a small fraction recognized bread as a contributor to overall salt consumption [39]. This limited awareness may have reduced the consumer-driven demand for lower-salt bread products, thereby diminishing pressure on producers to reformulate, despite initiatives specifically aimed at encouraging consumers to seek out and prefer bread with reduced salt content. The study evaluated a voluntary act, promoted via press releases, information on websites and infographics, encouraging bakers to adhere to the MoU. The assessment showed that although the Hellenic Federation of Bakers accepted the decision to reduce salt, the actual bakers did not adhere, potentially due to fear of non-consumer acceptance. Studies have shown that in order to achieve actual salt reductions in foods, mandatory regulations are required. Specifically, at least from a voluntary approach, working with the industry has shown some promise in salt reduction [40]; although the highest reduction was achieved in South Africa, they established a mandatory act in 2017, which has achieved ~1.2 g salt reduction in young adults per day. Similar challenges in lowering the salt content in bread were also reported in Iran, according to a qualitative retrospective policy analysis [41].

White bread in 2024 appeared to have a higher salt content when compared to brown bread: a trend that was also evident in 2012. This aligns with the findings from other studies in Slovenia [36] and Italy [20]. Generally, our study demonstrated substantial variation in salt content, even within similar bread types across both sampling periods, suggesting that salt reduction is technically feasible across all bread categories. Significant variability in the salt content of comparable bread products has been reported in other surveys too [20,42,43,44]. The high variability is often attributed to the absence of clear and enforceable legislation [45]: a factor that may be pertinent in the context of Greece. Specifically, while the Greek Food and Drink Code (national legislation) [46] sets an upper limit for salt in bread at 1.5%, it does not specify whether this threshold applies to bread “as consumed” or “on a dry matter basis”, thus limiting its effectiveness in guiding producers’ compliance.

Salt reduction in bread is generally a straightforward procedure and an effective strategy that can be easily implemented with minimal impact on product quality and consumer acceptability. Multiple studies have demonstrated that gradual salt reductions, ranging from 10% to over 50%, often go undetected by consumers and do not compromise sensory attributes such as flavor, texture, or overall liking [15,18,47,48,49]. For example, wheat breads produced with salt levels as low as 0.3 g/100 g maintained a comparable quality to those with typical salt concentrations (~1.2%) [21,50]. Technological properties were also unaffected by reductions of 0.3–0.6% salt [50,51]. Gradual stealth salt reduction strategies enable consumer taste adaptation, as shown in studies from Mozambique [45] and Tunisia [18], supporting salt decreases around 30–40% as being achievable without negatively impacting purchase intent or sensory acceptance [52,53,54]. Overall, the gradual reduction from approximately 1.4% (which is the current average salt content of artisanal bread in Greece) to 0.9–1.0% salt content in bread without substitutes (per the WHO Global Sodium Benchmarks) is both a feasible and advisable public health strategy to reduce sodium intake.

Bread consumers in Greece with salt intakes of less than 5 g per day from foods only (i.e., excluding the use of discretionary salt) has been projected to be 44.9% of the total Greek population (2021 census, *n* = 10,482,487) [55]. According to our modeling scenario 1, if a 1.2% salt upper level was implemented by all bakeries (as it was set in MoU), an additional 3.2% of Greek bread consumers who are currently exceeding 5 g of salt per day from food (*n* = 129,697) would reduce their total daily salt intake to below 5 g. Similarly, if the WHO sodium benchmark was adopted as an upper limit, our model showed that an extra 6.3% of baseline exceeders (*n* = 254,237) would achieve intakes of less than 5 g per day.

Standardizing salt levels can be effectively achieved through legislation, resulting in sustainable long-term benefits. Voluntary salt reduction initiatives, while often easiest to implement, frequently face significant resistance from manufacturers due to concerns about reduced sales, negative impacts on palatability and shelf life [45,51]. Despite awareness campaigns, incentives such as logos, stakeholder engagement and voluntary efforts often fail to motivate widespread reformulation [10,15], as our findings also demonstrate. The UK’s voluntary salt reduction program initially showed success, but lost momentum after governmental changes [56]. Interestingly, a recent report published by the Authority of the House of Lords in the UK has stated that voluntary reformulation programs largely failed, urging the UK Government to proceed with legislative measures [57]. Similarly, a systematic review indicated that between 2014 and 2019, countries that implemented mandatory maximum salt limits increased from nine in 2014 to nineteen in 2019, showing a trend towards regulatory approaches [58]. Half of these countries set mandatory maximum salt thresholds solely for bread [58]. However, in the EU, legislative salt limits in bread, as proposed in Portugal, face regulatory challenges, including free EU trade objections that hinder implementation [59], despite the fact that such approaches could substantially decrease salt consumption and save lives. Our work offers valuable, evidence-based insights that may serve as a basis for informed policy discussions about the increasingly recognized importance of mandatory salt thresholds in staple foods on both European and international levels.

### Limitations of the Study

There are some limitations that should be considered. Although the primary aim of this work was not to compare the salt content of bread between two sampling periods, but to evaluate the effectiveness of voluntary salt reduction initiatives, we acknowledge that the 2024 sampling period covered a broader geographical area than that of the 2012 period. The broader 2024 sampling, covering 87.5% of Greek regions, may have affected direct comparability with 2012, when only 38.5% of the regions was covered. To address this, a sensitivity analysis was performed solely using data from the two prefectures included in both years, with results indicating that the estimate is not biased by the broader 2024 sampling. Specifically, the fact that the salt content increased significantly in both full samplings and geographically matched subsets strengthens our confidence in our findings. Furthermore, increases in salt content were observed in both white and brown bread, supporting the generalizability of our conclusions across different bread types. Secondly, samples from the two periods were analyzed using different but accredited methods, in separate accredited laboratories participating in official food control programs in Greece. While this may have introduced some degree of inter-laboratory and inter-method variability, such variation is inherent to studies investigating temporal trends in food composition and does not compromise the validity of our overall conclusions. Finally, the modeling scenarios were based on the only available national food consumption data, which are more than a decade old. Hence, these data may not accurately reflect the current salt intake from bread, particularly given that there has been a drop in bread sales during the past decade. However, in such models, a baseline trend is selected (in this case, set as those collected in 2015), to have comparative results for specific intakes. The aim was to assess the potential impact of setting different sodium benchmarks in bread, rather than to record the actual current intake of salt from bread. Recent consumption data would be valuable to compare the predicted vs. actual consumption, but the increase in salt intake, if the consumption remained the same, is certain.

## 5. Conclusions

The findings highlight the failure of Greece’s voluntary national initiative to reduce salt in non-prepackaged artisanal bread, underscoring the limited impact of non-binding public health interventions. Moreover, the current average salt content in bread produced in Greece remains well above the WHO global sodium benchmark. Future research into the social, economic, and policy-level influences that affect salt use in bread are warranted to inform more effective public health interventions. However, a mandatory upper salt limit in non-prepackaged artisanal bread with robust monitoring would represent a more reliable and sustainable strategy for reducing salt content. Unlike voluntary efforts, legislation does not rely on consumer behavior changes through large and costly awareness campaigns or sustained industry motivation, overcoming barriers including information fatigue and uneven manufacturer compliance. Our results highlight the need for further policy dialog about implementing mandatory salt limits in staple foods such as bread on European and international levels.

## Figures and Tables

**Table 1:** Mean salt content (g/100 g) per broad category related to type of flour used (white, brown) in 2012 ^1^ and 2024 and percentage change.

Βread Type	Sampling 2012 ^1^		Sampling 2024		Percent Change in Mean ^2^
	*n* = 220	Mean (sd) (g/100 g)	*n* = 253	Mean (sd) (g/100 g)	
White	140	1.37 (0.24)	139	1.45 (0.23)	5.8
Brown	80	1.25 (0.36)	114	1.36 (0.36)	8.1

^1^ Data obtained from previous research results published in EFET website [1]. ^2^ Calculated using the formula: $\%change=\frac{Mean2024-Mean2012}{Mean2012}\times 100$ .

**Table 2:** Projected effects of salt reduction in artisanal bread on daily salt intake and prevalence of excessive salt consumption relative to WHO threshold for salt intake of 5 g/day.

Scenario	Median (p25, p75) Daily Salt Intake (g) from Artisanal Bread Only	Median (p25, p75) Daily Total Salt Intake (g) from All Foods	Consumers Exceeding WHO Salt Intake Threshold-Exceeders (*n*)	Percentage of Exceeders over Bread Consumers (*n* = 3127)	Percent Reduction in Exceeders Among Bread Consumers Overall (%)	Percent Reduction in Exceeders Relative to Baseline Exceeders (%)
Baseline analysis	0.49 (0.27, 0.80)	5.31 (3.69, 7.49)	1724	55.13		
Counterfactual scenario 1 All products ≤ 1.2 g/100 g bread	0.39 (0.22, 0.65)	5.18 (3.58, 7.36)	1669	53.37	1.7	3.2
Counterfactual scenario 2 All products ≤ 0.925 g/100 g bread (WHO benchmark)	0.31 (0.18, 0.50)	5.09 (3.49, 7.22)	1616	51.68	3.4	6.3

## Data Availability

The data presented in this study are available on request from the corresponding author due to legal restrictions.

## Supplementary Materials

The following supporting information can be downloaded at: https://www.mdpi.com/article/10.3390/nu17213374/s1. Table S1: Bread types classification and mean and minimum salt content (g/100 g) by specific bread type sampled in 2024. Table S2: Artisanal bread consumption in g using various quantification scenarios. Table S3: Distribution (%) of artisanal bread samples by salt content range in 2012 and 2024, and relative percent change from 2012 to 2024. Table S4. Baseline characteristics of artisanal bread consumers overall and by age group (children & adolescents, <19 years|adults, ≥19 years & <65 years|elderly, ≥65 years). Table S5: Median daily salt intake (g) in 2012 and 2024, overall and restricted to the two prefectures sampled in both years (Attica and Epirus).
