# Supplementary material for: Voluntary Food Reformulation Initiatives Failed to Reduce the Salt Content of Artisanal Breads in Greece
Source: Nutrients. 2025 Oct 27;17(21):3374. doi: 10.3390/nu17213374 (PMC12610621; doi:10.3390/nu17213374)
Supplement: Supplementary file 1 [file nutrients-17-03374-s001.zip › nutrients-3932983-supplementary.pdf]

Table S1. Bread types classification and mean and minimum salt content (g/100 g) by specific bread type sampled in 2024.

Broad category	n	%	EFET category[1]	n	%	Bread type	n	%	Mean salt content (SD) 2012 (g/100g)[1]	Mean salt content (SD) 2024 g/100g	Min salt content (SD) g/100g
White bread	114	45.06	White-rustic bread	66	26.1	Lagana	1	0.4	1.41	1.34	1.34
						Rustic whole-wheat bread	65	25.7		1.47 (0.20)	0.93
			White bread	73	28.8	Corn bread	4	1.6	1.3	1.58 (0.42)	1.15
						Refined wheat bread	66	26.1		1.42 (0.24)	0.88
						Bread with sourdough	1	0.4		1.49 (-)	1.49
						“Handmade” bread	1	0.4		1.42 (-)	1.42
						Bread with low glycemic index	1	0.4		1.17 (-)	1.17
Brown bread	139	54.94	Multi-cereal bread	46	18.2	Multi-cereal bread	46	18.1	0.92	1.37 (0.35)	0.54
			Rye bread	20	7.9	Rye-bread	20	7.9	1.24	1.40 (0.27)	0.64
			Whole-wheat bread	48	19.0	Dinkel	1	0.4	1.28	0.87 (-)	0.87
						Bread with no salt	1	0.4		0.08 (-)	0.08
						German type bread	1	0.4		1.27(-)	1.27
						Zea bread	1	0.4		1.24 (-)	1.24
						Walnut bread	1	0.4		1.94	1.94
						Whole wheat bread	43	17.0		1.36 (0.36)	0.01

[1] Data obtained from previous research results published in EFET website[1,21].

Table S2. Artisanal bread consumption in g using various quantification scenarios.

Quantification scenario	median	p25	p75	p95
1. Based only on 2 x24h recalls	33.0	19.8	55.0	118.0
1.1 With frequency of refined or whole wheat bread consumption	30.0	12.5	67.5	228.0
1.2 With average frequency of refined and whole wheat bread consumption	29.5	13.0	65.0	195.8
1.3 With cumulative frequency of refined and whole wheat bread consumption	37.2	17.0	80.6	256.6
2. Based only on FPQ data	34.3	25.7	77.0	147.9

Table S3. Distribution (%) of artisanal bread samples by salt content range in 2012 and 2024, and relative percent change from 2012 to 2024.

Range (g/100g bread)	2012 (%)	2024 (%)	Percent change
<0.5	1.4	0.8	-42.9
0.5-1	9.5	8.3	-12.6
1.01-1.2	20.9	10.3	-50.7
1.21-1.5	44.6	44.2	-0.9
1.51-2	22.7	34.8	53.3
>2.01	0.9	1.6	77.8

Table S4. Baseline characteristics of artisanal bread consumers overall and by age group (children & adolescents, <19 years | adults, ≥19 years & <65 years | elderly, ≥65 years).

Variables	Artisanal bread consumers, n=3127	Age groups			*p* for differences	*p*-trend
		Children & adolescents, <19 years, n=491	Adults, ≥19 years & <65 years, n=2145	Elderly, ≥65 years, n=491		
Age (years), median (25th, 75th percentile)	36 (24, 56)	9 (5, 14)	36 (27, 50)	73 (68, 80)	<0.001	<0.001
Sex, n (%)					<0.001	0.0299
Females	1729 (55.4)	230 (47.7)	1221 (56.9)	278 (56.6)		
Males	1389 (44.6)	252 (42.3)	924 (43.1)	213 (43.4)		
Daily bread consumption,	33 (19.8, 55.0)	28.8 (13.5, 50.0)	33 (19.1, 55.0)	37.5 (25.0, 66.0)	<0.001	<0.001

Variables	Artisanal bread consumers, n=3127	Age groups			p for differences	p-trend
		Children & adolescents, <19 years, n=491	Adults, ≥19 years & <65 years, n=2145	Elderly, ≥65 years, n=491		
median (25th, 75th percentile)						
Daily salt intake from bread, median (25th, 75th percentile)	0.49 (0.3, 0.8)	0.41 (0.2, 0.7)	0.48 (0.3, 0.8)	0.55 (0.3, 1)	<0.001	<0.001
Total daily salt intake level (excluding non-discretionary use of salt), n (%)					<0.001	<0.001
<5g	1403 (44.9)	248 (50.5)	828 (38.6)	327 (66.6)		
>=5g	1724 (55.1)	243(49.5)	1317 (61.40	164 (33.4)		
Total energy intake (kcal/day), mean(sd)	1919.9 (884.8)	1783.3 (835.6)	2055 (891.7)	1466.4 (715.6)	<0.001	<0.001
MedDiet score, mean(sd)	29.2 (6.2)		28.9 (6.3)	30.5 (5.8)	<0.001	

Table S5. Median daily salt intake (g) in 2012 and 2024, overall and restricted to the two prefectures sampled in both years (Attica and Epirus).

Year	Prefectures	Region's coverage	Median (p25, p75) salt intake (g)
2012	Attica & Epirus only	38.5%	0.44 (0.25, 0.73)
2024	Attica & Epirus only	38.5%	0.47 (0.24, 0.78)
2024	Nine (9) prefectures	87.5%	0.49 (0.27, 0.80)
